# Facial Paralysis as Initial Manifestation of Light-chain Amyloidosis

**DOI:** 10.7759/cureus.5521

**Published:** 2019-08-29

**Authors:** André M Martins, Filipa S Ferreira, Ines M Leite, Marina Fonseca, Rui Victorino

**Affiliations:** 1 Internal Medicine, Serviço De Medicina 2, Hospital De Santa Maria, Lisboa, PRT

**Keywords:** al amyloidosis, peripheral facial paralysis, heart failure, cardiac amyloidosis, cranial nerve amyloidosis

## Abstract

Light-chain (AL) amyloidosis is a systemic disease capable of damaging virtually all body tissues. Neurologic involvement is commonly manifested by dysautonomia and peripheral nervous system affection. However, from 1970 to 2018, only 12 cases of cranial nerve injury associated with AL amyloidosis were identified.

Eight months before hospital admission, a previously healthy 61-year-old man complained to his general practitioner of episodes of lipotimia while walking and, three months later, he developed a left facial nerve paralysis assumed, at that time, to be idiopathic. After two months, he started complaining of dyspnea and lower limb edema. Physical examination at admission revealed hypotension, exuberant peripheral edema, jugular venous distention, periorbital purpura and left peripheral facial paralysis. He had elevated troponin and brain natriuretic peptide, mild proteinuria and a monoclonal gammopathy IgG/lambda. Bone marrow biopsy revealed 20% plasmocytes and cardiac ultrasound showed diffuse hypokinesia and restrictive filling pattern. AL amyloidosis with major cardiac involvement was considered and a rectal biopsy revealed amyloid protein. Chemotherapy protocol to AL amyloidosis was initiated but cardiac disease progressed leading to death.

Persistent facial nerve paralysis should be considered as a rare initial manifestation of AL amyloidosis allowing an earlier diagnosis.

## Introduction

Light-chain (AL) amyloidosis is a hematologic disorder characterized by deposition in tissues of light-chain fibrils of immunoglobulin, most commonly of the lambda isotype, that cause organ damage. Although it is closely related to multiple myeloma, most patients have ≤20% of plasma cells in the marrow [1]. AL amyloidosis is the most common type of systemic amyloidosis, with an estimated incidence of nine cases/million inhabitant/year. The average age of diagnosed patients is 65 years and less than 10% are under 50 years old [2].

Clinical findings of AL amyloidosis include proteinuria with possible progression to nephrotic syndrome, hepatomegaly with elevated liver enzyme levels, macroglossia, purpura with periorbital distribution (raccoon eyes), bleeding diathesis, and, in advanced stages, right-sided heart failure due to restrictive cardiomyopathy [2]. Neurologic manifestations are also well recognized, usually presenting with the involvement of the peripheral nervous system with symptoms of numbness, paresthesia and pain, and dysautonomia with the occurrence of orthostatic hypotension [3]. Involvement of cranial nerves has been only occasionally reported [4]. In a literature review conducted on PubMed, from 1970 to 2018, only 12 cases of cranial nerve injury associated with AL amyloidosis were identified [4-11]. We report here a case of AL amyloidosis presenting initially with facial nerve paralysis.

## Case presentation

A previously healthy 61-year-old male patient was admitted with congestive heart failure. Eight months before the admission, he was evaluated for episodes of lipotimia manifested while walking. At this point, the patient had normal blood tests, except for an elevated erythrocyte sedimentation rate (ESR) of 51 mm/h. A chest radiography, a holter, and a cardiac ultrasound were also normal. Three months later, he presented with left peripheral facial paralysis which was assumed to be idiopathic, after excluding vascular, infectious, and autoimmune causes. Two months later, he complained of dyspnea during progressively shorter walks and developed lower limbs edema. These symptoms were aggravated in six weeks before admission to an Internal Medicine ward for investigation.

**Figure 1:**
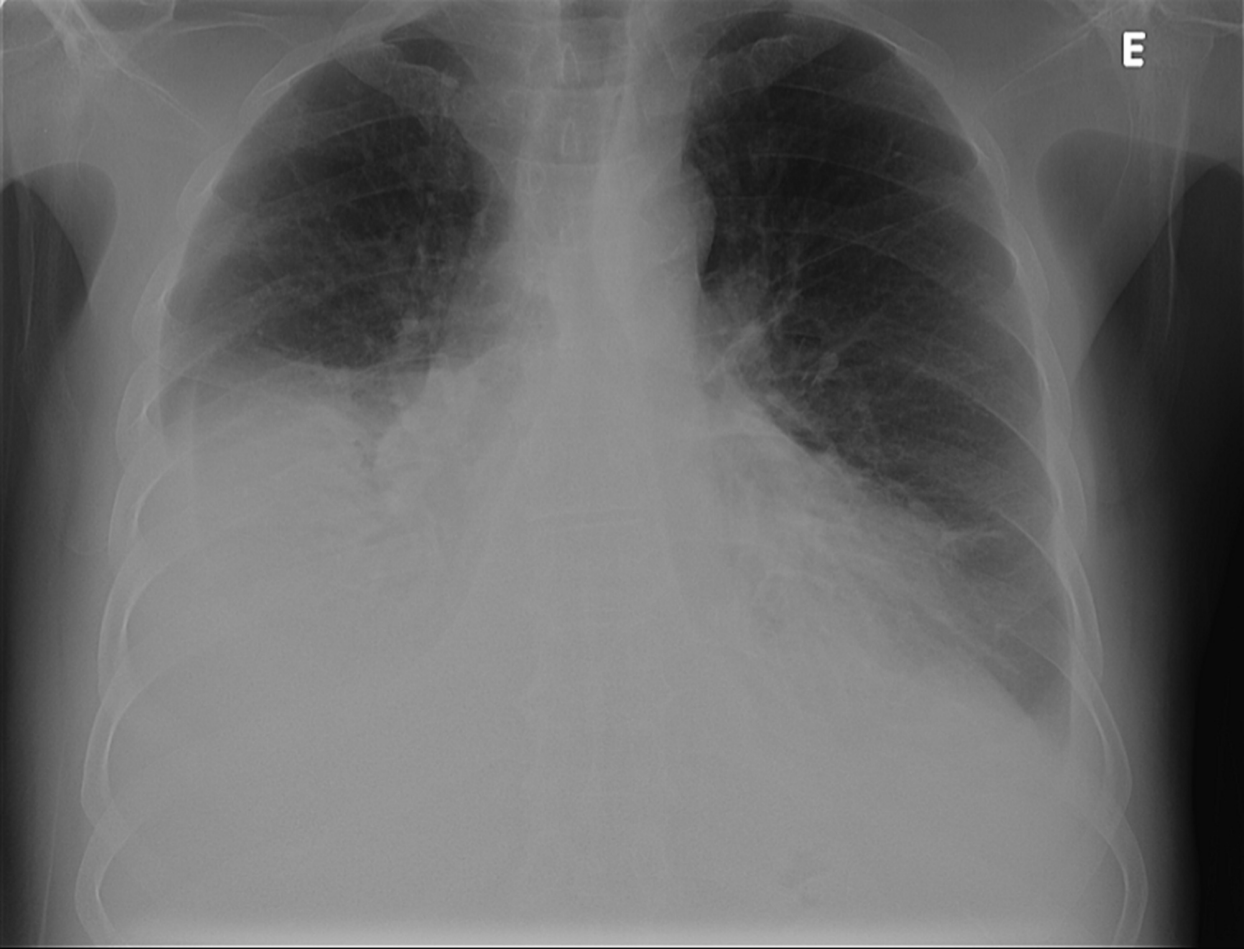
Chest radiography, posteroanterior view Chest radiography, posteroanterior view, on admission, showing voluminous right pleural effusion.

In the initial observation at the hospital, he presented with hypotension (94/58 mmHg), exuberant peripheral edema, jugular venous distention, periorbital purpura, and maintained left peripheral facial paralysis. Pulmonary auscultation and percussion were suggestive of voluminous right pleural effusion (Figure 1), confirmed by chest radiography which led to a diagnostic and therapeutic thoracentesis, with a drainage of 1.5 L of pleural liquid compatible with transudate.

Laboratory studies revealed an hemoglobin of 13.9 g/dL (normal range [NR]: 13.0-17.5), platelet count of 201x10^9^/L (NR: 150-450), elevated ESR of 72 mm/h, slight prolongation of prothrombin time of 14.0/11.6 seconds, mild renal failure (estimated creatinine clearance of 50 mL/min/1.73 with urea levels of 64 mg/dL [NR: 10-50]), elevated troponin T (0.086 ng/mL [NR: <0.014]), aspartate aminotransferase (100 U/L [NR: <34]), alanine aminotransferase (329 U/L [NR: 12-78]), alkaline phosphatase (290 U/L [NR: 44-147]), gamma-glutamyltransferase (551 U/L [NR: 0-40]), total bilirubin (1.8 mg/dL [NR: < 1.2]), and lactic dehydrogenase (630 U/L [NR: 100-250]). A marked elevation of brain natriuretic peptide (NT-BNP) (31,151 ng/mL [NR: < 900]) was also detected.

Serum protein electrophoresis showed a narrow gamma band of 1.5 g/dL. Additionally, immunofixation, immunoglobulin, and light-chain measurements documented a monoclonal gammopathy IgG/lambda (IgG 2,280 mg/dL [NR: 751-1,560], lambda 1,420 mg/dL [NR: 313-723], kappa 381 mg/dL [NR: 629-1,350, free kappa light chains 14.9 mg/L [NR: 3.3-19.4], free lambda light chains 186 mg/L [NR: 5.7-26.3], kappa/lambda ratio 0.08 [NR: 0.26-1.65]) with Bence-Jones lambda proteinuria (1,300 mg/24 h). A plasma cell dyscrasia was considered and a bone marrow biopsy was performed, which revealed an elevated proportion of plasmocytes (20%).

**Figure 2:**
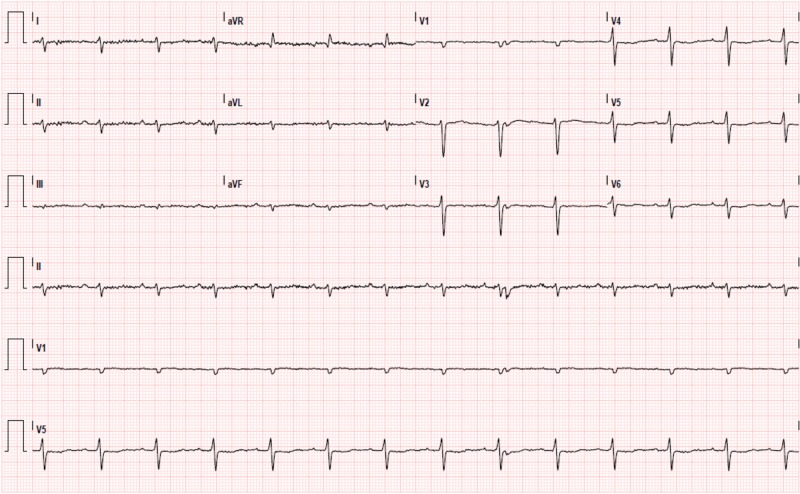
Electrocardiogram Electrocardiogram, on admission, showing low-voltage QRS complexes.

**Figure 3:**
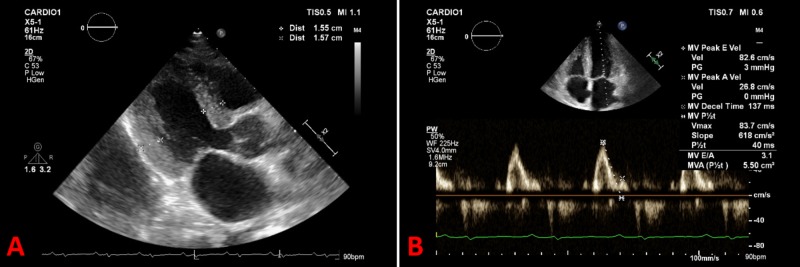
Cardiac ultrasound Cardiac ultrasound showing: A) left ventricular hypertrophy; B) restrictive left ventricular filling pattern with E/A ratio 3.1 and E/e' ratio 20.

The electrocardiogram showed low-voltage QRS complexes (Figure 2), and the cardiac ultrasound showed left ventricular hypertrophy with diffuse hypokinesia and mildly impaired systolic function (left ventricular ejection fraction 51%) with a restrictive filling pattern (Figure 3). AL amyloidosis with major cardiac involvement was considered, and, in order to confirm this diagnosis, a cardiac magnetic resonance was conducted and showed diffuse thickening of the myocardium with late gadolinium enhancement, consistent with amyloid deposition,and rectal biopsy revealed amyloid protein in blood vessel walls and subepithelium. The diagnosis of AL amyloidosis was thus established on the basis of immunologic studies revealing a monoclonal gammopathy IgG/lambda and biopsy findings. 

During the investigation, the patient initiated treatment for symptomatic heart failure with diuretics (furosemide) and ivabradine with slight symptom improvement. Angiotensin-converting enzyme inhibitor and beta-blocker were not tolerated due to hypotension.

A chemotherapy protocol for AL amyloidosis was initiated using cyclophosphamide, bortezomib, and dexamethasone (CyBorD), but cardiac disease progressed with a further elevation of NT-BNP (55,250 ng/mL) and the need of supplementary oxygen and vasopressor therapy, leading to death from cardiogenic shock two months after diagnosis.

## Discussion

This case illustrated well the classic cardiac manifestations of AL amyloidosis as well as its dysautonomic expression, and exhibited the particularity of an early cranial nerve involvement, namely peripheral facial paralysis, which is an extremely rare manifestation [4]. Peripheral facial nerve paralysis was attributed to nerve amyloid involvement considering that autoimmune, vascular, and infectious causes were excluded. Moreover, the patient fulfilled all the other criteria for AL amyloidosis, namely the presence of elevated plasmatic and urinary lambda light chains with increased bone marrow plasmocytes and documentation of amyloid protein in rectal biopsy.

AL amyloidosis is an underdiagnosed and rare cause of cranial neuropathies [4,5]. Out of the 12 cases of cranial nerve involvement associated with AL amyloidosis reviewed in the literature, facial nerve paralysis was described in seven cases [4,6-9]. In the remaining cases, the cranial nerves involved were the olfatory, oculomotor, trochlear, trigeminal, abducens, and hypoglossal [4-7,10,11]. Cranial nerve deficits usually preceded the diagnosis of AL amyloidosis. In our case, the facial peripheral paralysis presented six months before symptomatic heart failure and was the complaint that led to the initial etiologic investigation. In fact, some authors suggest that AL amyloidosis should be suspected when cranial neuropathy is associated with proteinuria or monoclonal gammopathy [4]. 

In the six cases described by Traynor et al. in which cranial neuropathy was a major manifestation of AL amyloidosis, three of the patients had multiple cranial nerves involvement and two had isolated peripheral facial nerve palsy [4]. The oculomotor, trochlear, trigeminal, and the facial cranial nerves were the most often affected in these cases. All had renal involvement manifested by proteinuria. Jeanning et al. reported a case of a hemorrhagic bullae at the palate and hypotrophy of the side of the tongue due to left hypoglossal paralysis which preceded the diagnosis of AL amyloidosis by two years [5]. Van Gerpen et al. described the first case of amyloidosis involving nasopharynx and multiple cranial nerves manifested by epistaxis [7]. In this patient, peripheral facial paralysis developed five years before diagnosis and monoclonal gammopathy and proteinuria were detected after two years. 

Although the patient initially reported orthostatic hypotension, the detailed cardiac and laboratory investigation at that time did not identify evidence of AL amyloidosis. The typical cardiac alterations appeared five months later, namely the presence of diffuse hypokinesia with preserved ejection fraction and diastolic dysfunction in cardiac ultrasound and the elevation of cardiac biomarkers, such as troponin T and NT-BNP. At the time of diagnosis, our patient was considered to be in stage III, because of elevated NT-BNP and troponin values with a difference between involved and uninvolved free light chains (dFLC) of 171 mg/dL, which is next to the proposed cutoff for stage IV (180 ng/mL). According to this staging system, the median overall survival from diagnosis was 14 months, which was more than what we observed in our case [12]. Although dFLC was not in the range of stage IV, NT-BNP was markedly elevated, reflecting the severe cardiac involvement, which is known to predict worse prognosis and, in this case, was responsible for the fatal outcome [1].

When the patient was admitted, there were also laboratory alterations compatible with amyloid deposition in the liver, as expected at this stage of the disease, although these changes could also be explained by hepatic congestion due to right-sided heart failure. In the 12 cases reviewed with cranial nerve involvement, three patients presented altered biochemical parameters of liver function and hepatic amyloid infiltration confirmed by biopsy [4,8,9].

## Conclusions

This case highlights the possibility of cranial nerve involvement, namely facial nerve paralysis, as an early manifestation of AL amyloidosis and should therefore be considered in the etiological investigation of a peripheral facial nerve paralysis.
